# Effects of phytase supplementation and increased nutrient density on growth performance, carcass characteristics, and hypothalamic appetitive hormone expression and catecholamine concentrations in broilers from 1 to 43 days of age^1^

**DOI:** 10.1016/j.psj.2021.101495

**Published:** 2021-09-21

**Authors:** R. Kriseldi, M.R. Bedford, R.N. Dilger, C.D. Foradori, L. MacKay, W.A. Dozier

**Affiliations:** ⁎ Department of Poultry Science, Auburn University, Auburn, AL 36849, USA; † AB Vista, Marlborough, Wiltshire, SN8 4AN, United Kingdom; ‡ Department of Animal Science, University of Illinois, Urbana, IL 61801, USA; § Department of Anatomy, Physiology, and Pharmacology, Auburn University, Auburn, AL 36849, USA

**Keywords:** broilers, phytase, inositol, dopamine, brain

## Abstract

Two experiments were conducted to evaluate extra-phosphoric effects of phytase and nutrient density on growth performance, meat yield, and hypothalamic appetitive hormone expression and catecholamine concentrations of broilers. Experiment 1 determined differences of digestible amino acid concentrations and AME_n_ using 256 Yield Plus × Ross 708 broilers (32 cages, 8 birds/cage) fed diets without or with 4,500 phytase units (**FTU**)/kg inclusion (16 reps/treatment). In Experiment 2, 832 Yield Plus × Ross 708 broilers (32 pens; 26 birds/pen) were provided diets in a 2 × 2 factorial arrangement consisting of 2 nutrient contents (without or with increased density) and 2 phytase inclusions (0 or 4,500 FTU/kg). Increased nutrient density was formulated to contain 0.007, 0.015, 0.013, 0.021, 0.024%, and 61 kcal/kg higher digestible SAA, Lys, Thr, Val, Ile, and AME_n_ (from Experiment 1) respectively, compared with the control diet. Growth performance was determined at 14, 28, and 40 d of age and carcass characteristics at 41 d of age. At 43 d of age, plasma inositol, hypothalamic appetitive hormone expression, and catecholamine concentrations were determined from 4 birds/pen. Additive effects of phytase inclusion and increased nutrient density resulted in the lowest (*P* < 0.05) feed conversion from 1 to 40 d of age and the heaviest (*P* < 0.01) breast meat weights among dietary treatments. Phytase addition numerically increased feed intake (*P* = 0.06) and BW gain (*P* = 0.051) compared with birds fed diets without phytase from 1 to 40 d of age. Plasma inositol and dopamine concentrations were 2.3- and 1.2-fold higher (*P* < 0.01), respectively, in broilers fed phytase-added diets than birds fed diets without phytase inclusion. However, mRNA expression of neuropeptide Y, agouti-related peptide, proopiomelanocortin, cholecystokinin A receptor, ghrelin, and serotonin concentration were not different (*P* > 0.05) among treatments. These data indicated additive effects of phytase supplementation and increased nutrient density on growth performance and meat accretion of broilers. However, the influence of phytase on feed intake warrants future research.

## INTRODUCTION

Phytase supplementation targeting effects beyond phosphorus liberation was reported to enhance growth performance and carcass characteristics of broilers (Gehring et al., 2013; Campasino et al., 2014; Walk et al., 2014; Beeson et al., 2017). Although these effects have been attributed to increased nutrient utilization (Selle and Ravindran, 2007; Selle et al., 2012; Dersjant-Li et al., 2015), benefits of extra-phosphoric effects of phytase may also originate from the stimulation of feed intake (Walk and Olukosi, 2019). A previous study in our laboratory demonstrated that increasing *E. coli-*derived phytase concentrations from 0 to 40,500 FTU/kg elicited a positive correlation (r = 0.73, *P* < 0.001) between feed intake and BW gain of broilers with maximum feed intake of broilers obtained with 4,500 FTU/kg inclusion (Kriseldi et al., 2019). Similarly, Walk and Olukosi (2019) reported that the increased BW gain of broilers due to *E. coli-*derived phytase addition of 0, 2,000, or 4,000 FTU/kg was better associated with increased digestible amino acid intake compared with amino acid digestibility alone, emphasizing the importance of intake in this response. Therefore, extra-phosphoric effects of phytase addition may include feed intake, which translates to increased nutrient intake (Walk and Olukosi, 2019).

Despite the potential benefits of phytase in increasing feed intake, research evaluating mechanisms of extra-phosphoric effects of phytase on nutrient intake is lacking. Presumably, changes in feeding behavior may be influenced by the effect of phytase on phytate degradation (Watson et al., 2006; Liu et al., 2014). Nutrients liberated from phytate hydrolysis may serve as indicators or precursors to alter mRNA expression of appetitive hormones or catecholamines, thereby leading to changes in feed intake (Richards and Proszkowiec-Weglarz, 2007; Dridi, 2017). With these considerations, 2 experiments were conducted to determine extra-phosphoric effects of phytase and increased nutrient density on growth performance, meat yield, and hypothalamic appetitive hormone expression and catecholamine concentrations of broilers throughout a 6-wk production period.

## MATERIALS AND METHODS

All procedures involving live birds were approved by Auburn University Institutional Animal Care and Use Committee (PRN 2018–3394). Two experiments were conducted utilizing Yield Plus × Ross 708 male chicks (Aviagen North America, Huntsville, AL). Chicks were obtained from a commercial hatchery post-hatch and were vaccinated for Marek's disease, Newcastle disease, and infectious bronchitis.

### Experiment 1

#### Bird Husbandry

Two hundred and fifty-six broiler chicks were placed into 32 battery cages (8 birds/cage; 0.06 m^2^/bird; Petersime, Gettysburg, OH). Cages were placed in a solid-sided room equipped with exhaust fans, forced-air heaters, and evaporative coolers to adjust room temperature. Each cage was provided with 1 trough feeder and 1 trough waterer. The temperature of the room was set at 33°C at placement and was gradually decreased to 25°C at 23 d of age. Photoperiod was provided at 23L:1D from 1 to 7 d of age and 20L:4D from 8 to 23 d of age, while light intensity was maintained at 30 lux throughout the experimental period. Feed and water were provided ad libitum.

#### Dietary Treatments

Two dietary treatments were fed from 1 to 23 d of age consisting of a control diet and a diet supplemented with 4,500 phytase units (**FTU**)/kg (Table 1). Treatment 1 was formulated with calcium and non-phytate phosphorus at 0.96 and 0.48%, respectively. Treatment 2 was formulated similar to Treatment 1 but with 0.165 and 0.150% points of calcium and non-phytate phosphorus, respectively, originated from phytase supplementation. Phytase was supplemented in Treatment 2 at 4,500 FTU/kg. *Escherichia coli*-derived phytase expressed in *Trichoderma reesei* was supplemented in Treatment 2 at 4,500 FTU/kg (Quantum Blue 5G, AB Vista, Marlborough, UK). One FTU was defined as the quantity of phytase required to release 1 µmol of inorganic phosphate from 0.0051 mol/L sodium phytate in 1 min at pH 5.5 and 37°C. Phytase concentration was selected based on a previous study in our laboratory, which indicated that optimum growth performance of broilers may be obtained by supplementing phytase at 4,500 FTU/kg (Kriseldi et al., 2019). Both treatments were formulated with digestible amino acid concentrations at 93% of Aviagen Ross 708 Broiler Nutrition Specifications (Aviagen, 2016) to mimic moderate amino acid specifications used in the US broiler industry.

**Table 1:** Ingredient and nutrient composition of dietary treatments fed to broilers from 1 to 23 D of age, Experiment 1.

	Dietary treatment	
Ingredient, %	1	2
Corn	57.94	59.60
Soybean meal (46%)	35.93	35.62
Vegetable oil	1.15	0.54
Dicalcium phosphate	2.04	1.23
Limestone	1.05	1.08
Sodium chloride	0.39	0.32
DL-Met	0.30	0.30
L-Lys•HCl	0.19	0.20
L-Thr	0.12	0.12
Titanium dioxide	0.50	0.50
Builder sand	0.10	0.10
Mineral premix^1^	0.10	0.10
Vitamin premix^2^	0.10	0.10
Choline chloride	0.07	0.07
Xylanase^3^	0.01	0.01
Phytase^4^	—	0.11
Calculated nutrient composition, % (unless otherwise noted)		
AME_n_, kcal/kg	3,000	3,000
Crude protein	21.54	21.54
Digestible Lys	1.19	1.19
Digestible Met	0.60	0.60
Digestible SAA	0.88	0.88
Digestible Thr	0.80	0.80
Digestible Val	0.89	0.89
Digestible Ile	0.81	0.81
Ca	0.96	0.80
Non-phytate P	0.48	0.33
Na	0.18	0.18
Analyzed nutrient composition, % (unless otherwise noted)		
Phytase activity, FTU/kg^5^	85	3,690
Crude protein	21.42	21.75
Ca	1.12	0.83
Total P	0.83	0.59

1 Trace mineral premix include per kg of diet: Mn (manganese sulfate), 120 mg; Zn (zinc sulfate), 100 mg; Fe (iron sulfate monohydrate), 30 mg; Cu (tri-basic copper chloride), 8 mg; I (ethylenediamine dihydriodide), 1.4 mg; and Se (sodium selenite), 0.3 mg.

2 Vitamin premix includes per kg of diet: Vitamin A (Vitamin A acetate), 18,739 IU; Vitamin D_3_ (cholecalciferol), 6,614 IU; Vitamin E (DL-alpha tocopherol acetate), 66 IU; menadione (menadione sodium bisulfate complex), 4 mg; Vitamin B_12_ (cyanocobalamin), 0.03 mg; folacin (folic acid), 2.6 mg: D-pantothenic acid (calcium pantothenate), 31 mg; riboflavin (riboflavin), 22 mg; niacin (niacinamide), 88 mg; thiamin (thiamin mononitrate), 5.5 mg; biotin (biotin), 0.18 mg; and pyridoxine (pyridoxine hydrochloride), 7.7 mg.

3 Econase XT, AB Vista Feed Ingredients, Marlborough, UK.

4 *Escherichia coli-*derived phytase from *Trichoderma reesei* (Quantum Blue 5G, AB Vista Feed Ingredients, Marlborough, UK).

5 One unit of phytase activity (FTU) is the quantity of enzyme to liberate 1 µmol of inorganic phosphate per minute from 0.0051 mol/L sodium phytate at pH 5.5 and 37°C.

#### Measurements, Sample Collection, Chemical Analysis, and Calculations

Birds and feed were weighed at 1 and 19 d of age to determine BW, BW gain, feed intake, and feed conversion ratio (**FCR**). The incidence of mortality was recorded daily. From 20 to 22 d of age, feed consumption and excreta output were measured for determination of AME_n_ of dietary treatments. Excreta subsamples were collected from 10 locations of the pan beneath each cage and homogenized to create a pooled sample (approximately 2 kg) for each pen. Representative samples of excreta and feed were lyophilized in a freeze dryer (VirTis Genesis 25ES, SP Industries Inc., Warminster, PA). Dried samples were ground through a coffee grinder to ensure a homogenous mixture. Duplicate 0.8 g samples of feed and excreta were analyzed for gross energy using an isoperibol oxygen bomb calorimeter (Model no. 6300, Parr Instruments, Moline, IA), while nitrogen content (250 mg samples) was measured using a combustion analyzer (Rapid N cube, Elementar, Hanau, Germany) according to Dumas method (method 968.06; AOAC International, 2006). Nitrogen-corrected AME for each dietary treatment was calculated using a nitrogen correction factor of 8.73 kcal/g (Titus, 1956) with the following equation:

$$AME_{n}=\frac{\left[GE_{Intake}\left(kcal\right)-GE_{Excretion}\left(kcal\right)\right]-\left\{8.73\left(kcal/g\right)\;\times \left[N_{Intake}\left(g\right)-N_{Excretion}\left(g\right)\right]\right\}}{Feed\;Intake\;\left(kg\right)}$$

where, GE_Intake_ and GE_Excretion_ represent the amount of gross energy consumed and excreted, respectively; and N_Intake_ and N_Excretion_ represent the amount of nitrogen consumed and excreted by broilers.

At 23 d of age, ileal digesta from 6 birds per cage was collected to determine the apparent ileal amino acid digestibility of dietary treatments. Ileal digesta from each bird was collected by gently flushing the terminal ileum of broilers using deionized-distilled water, which was defined as the terminal one-third of the section between the Meckel's diverticulum and approximately 4 cm anterior from the ileocecal junction (Rodehutscord et al., 2012). Pooled ileal digesta contents were immediately frozen by submersion in liquid nitrogen for approximately 5 min to terminate phytase activity. Samples were kept on ice until further analysis.

Ileal digesta samples were lyophilized (VirTis Genesis 25ES, SP Industries Inc.) and ground with an electric coffee grinder. Samples were analyzed for total amino acid concentrations in duplicate at Ajinomoto Heartland LLC using ion exchange chromatography with postcolumn ninhydrin derivatization. Hydrolysates were obtained using acid hydrolysis for all amino acids except for Met, Cys, and Trp, performic acid oxidation followed by acid hydrolysis for Met and Cys, and alkaline hydrolysis for Trp [method 982.30 E(a,b,c); AOAC International, 2006].

Analysis of titanium dioxide content in feed samples was conducted in quadruplicate (600 mg), while ileal digesta samples were analyzed in duplicate (200 mg) (Short et al., 1996). Samples were ashed for 12 h at 580°C in porcelain crucibles. After ashing, samples were rinsed into a 50-mL beaker using 10 mL of sulfuric acid (7.4 M). The solutions were heated at 250°C on a hot plate to dissolve solid particles for approximately 60 min. After cooling to room temperature, solutions were rinsed using 10 mL distilled water into a glass beaker containing 25 mL distilled water. Twenty mL of hydrogen peroxide (30%) was added to each beaker and the solution was diluted to 100 mL with distilled water. Solutions were kept at room temperature for at least 48 h prior to absorbance measurement at 410 nm using a spectrophotometer (SpectraMax Plus 384, Molecular Devices LLC., San Jose, CA). Titanium concentration was determined by comparing absorbance results with known standards using a linear least-squares regression. Concentrations of total amino acids and titanium dioxide from feed and ileal digesta analyses were used to calculate apparent ileal amino acid digestibility using the following equation:

$$Apparent\;ileal\;amino\;acid\;digestibility=\;\left[1-\left(\frac{TiO_{2\;Diet}}{TiO_{2\;Digesta}}\right)\times \left(\frac{AA_{Digesta}}{AA_{Diet}}\right)\right]\times \;100$$

where TiO_2 Digesta_ and TiO_2 Diet_ represent the analyzed concentrations of titanium dioxide in the ileal digesta and diets, respectively, and AA_Digesta_ and AA_Diet_ indicate the analyzed total amino acid concentrations in ileal digesta and diets, respectively.

#### Statistical Analysis

This experiment was conducted as a randomized complete block design with cage location as the blocking factor. Individual cage represented the experimental unit with 16 replications. Data were subjected to 1-way ANOVA and statistical significance was considered at *P* ≤ 0.05.

### Experiment 2

#### Bird Husbandry and Processing

Eight hundred and thirty-two broiler chicks were placed into 32 floor pens (26 birds/pen; 0.08 m^2^/bird) equipped with a tube feeder, a nipple drinker line, and litter (from 2 previous flocks). Birds were housed in a solid-sided house with a negative-pressure ventilation system equipped with exhaust fans, cooling pads, and forced-air heaters to adjust house temperature. At placement, ambient temperature was set at 33°C and was gradually decreased to 20°C at 41 d of age. Light intensity was maintained at 30, 10, and 5 lux from 1 to 7, 8 to 14, and 15 to 41 d of age, respectively. Photoperiod was provided at 23L:1D from 1 to 7 d of age and 20L:4D for the remainder of the experiment. Feed and water were provided ad libitum. Feed and birds were weighed at 1, 14, 28, and 40 d of age to determine BW, BW gain, feed intake, and FCR. The incidence of mortality was recorded daily.

At 41 d of age, 14 birds per pen were selected for processing. Feed was removed from each pen 10 h prior to processing. Selected birds were placed in coops and transported to the Auburn University Pilot Processing Plant. Broilers were hung on shackles, electrically stunned, slaughtered, scalded, picked, and manually eviscerated. Following 3 h of chilling in an ice bath, carcass (without abdominal fat) and abdominal fat weights were recorded. Whole carcasses were then placed in ice for 18 h. The whole carcass was deboned to determine weights of *pectoralis major* muscle (boneless breast) and *minor* muscle (tenders), wing, drum, and thigh. Meat yields were calculated relative to the 40-d live BW.

#### Dietary Treatments

Four dietary treatments were provided for starter (from 1 to 14 d of age), grower (from 15 to 28 d of age), and finisher (from 29 to 43 d of age) periods (Table 2). Experimental diets were provided in a 2 × 2 factorial arrangement consisting of 2 nutrient contents (without or with increased nutrient density) and 2 phytase inclusions (0 or 4,500 FTU/kg). Treatment 1 was the control diet. Treatment 2 was formulated with increased nutrient density. Treatment 3 was formulated to contain phytase addition at 4,500 FTU/kg. Treatment 4 was formulated with both increased nutrient density and phytase supplementation at 4,500 FTU/kg. Diets with increased nutrients density were formulated to contain higher digestible SAA, Lys, Thr, Val, Ile concentrations, and AME_n_ at 0.007, 0.015, 0.013, 0.021, 0.024%, and 61 kcal/kg, respectively. Increased nutrient density was obtained from differences in digestible amino acid concentrations and AME_n_ between the control and the diet with phytase addition in the Experiment 1. Increased nutrient density was designed to mimic the effects of phytase supplementation on nutrient intake. Additionally, phytase concentration was selected based on a previous study in our laboratory, which indicated that optimum growth performance may be obtained when broilers were supplemented with 4,500 FTU/kg phytase (Kriseldi et al., 2019). Phytase addition provided 0.165 and 0.150%-points of calcium and non-phytate phosphorus. Phytase used was *E. coli-*derived phytase expressed in *Trichoderma reesei* (Quantum Blue 5G, AB Vista. All treatments were formulated to contain 93% amino acid concentrations of Aviagen Ross 708 Broiler Nutrition Specifications (Aviagen, 2016) to mimic moderate amino acid specifications used in the US broiler industry. Feed samples from each experiment were analyzed for phytase activity by ELISA specific for Quantum Blue (ESC, Standard Analytical Method, SAM099; AB Vista) similar to the method described by Engelen et al. (2001).

**Table 2:** Ingredient and nutrient composition of dietary treatments fed to broilers, Experiment 2.

	Starter, 1 to 14 D				Grower, 15 to 28 D				Finisher, 29 to 43 D			
Ingredient, %	1	2	3	4	1	2	3	4	1	2	3	4
Corn	60.01	56.53	61.58	58.10	64.23	60.75	66.28	62.80	67.84	64.36	69.88	66.41
Soybean meal (46%)	34.75	36.58	34.51	36.35	30.02	31.85	29.71	31.54	25.43	27.26	25.12	26.96
Vegetable oil	0.73	2.43	0.14	1.84	1.64	3.34	0.87	2.57	2.50	4.20	1.73	3.43
Dicalcium phosphate	2.18	2.18	1.37	1.36	1.97	1.96	0.91	0.91	1.76	1.75	0.70	0.69
Limestone	0.98	0.97	1.01	1.00	0.90	0.89	0.94	0.93	0.82	0.81	0.86	0.85
Salt	0.39	0.39	0.32	0.32	0.40	0.40	0.30	0.30	0.40	0.40	0.30	0.30
DL-Met	0.31	0.31	0.31	0.31	0.28	0.27	0.27	0.27	0.25	0.25	0.24	0.24
L-Lys•HCl	0.23	0.20	0.23	0.20	0.21	0.19	0.22	0.19	0.20	0.17	0.20	0.17
L-Thr	0.13	0.12	0.12	0.12	0.10	0.10	0.10	0.09	0.08	0.07	0.08	0.07
Mineral premix^1^	0.10	0.10	0.10	0.10	0.10	0.10	0.10	0.10	0.10	0.10	0.10	0.10
Vitamin premix^2^	0.10	0.10	0.10	0.10	0.08	0.08	0.08	0.08	0.05	0.05	0.05	0.05
Choline chloride	0.07	0.07	0.07	0.07	0.07	0.07	0.07	0.07	0.07	0.07	0.07	0.07
Xylanase^3^	0.01	0.01	0.01	0.01	0.01	0.01	0.01	0.01	0.01	0.01	0.01	0.01
L-Val	0.01	0.01	0.01	0.01	—	—	—	—	—	—	—	—
Titanium dioxide	—	—	—	—	—	—	—	—	0.50	0.50	0.50	0.50
Phytase^4^	—	—	0.11	0.11	—	—	0.14	0.14	—	—	0.14	0.14
Calculated nutrient composition, % (unless otherwise noted)												
AME_n_, kcal/kg	3,000	3,061	3,000	3,061	3,100	3,161	3,100	3,161	3,180	3,241	3,180	3,241
Crude protein	21.47	22.01	21.49	22.03	19.57	20.10	19.60	20.13	17.69	18.23	17.72	18.25
Digestible Lys	1.19	1.21	1.19	1.21	1.07	1.09	1.07	1.09	0.95	0.96	0.95	0.96
Digestible Met	0.60	0.61	0.60	0.60	0.55	0.55	0.54	0.55	0.50	0.50	0.50	0.50
Digestible SAA	0.88	0.89	0.88	0.89	0.81	0.82	0.81	0.82	0.74	0.75	0.74	0.75
Digestible Thr	0.80	0.81	0.80	0.81	0.72	0.73	0.72	0.73	0.63	0.65	0.63	0.65
Digestible Val	0.89	0.91	0.89	0.91	0.81	0.83	0.81	0.83	0.74	0.76	0.74	0.76
Digestible Ile	0.80	0.82	0.80	0.82	0.73	0.75	0.73	0.75	0.65	0.68	0.65	0.68
Digestible Arg	1.29	1.34	1.29	1.34	1.16	1.20	1.16	1.20	1.03	1.07	1.03	1.07
Ca	0.96	0.96	0.80	0.80	0.87	0.87	0.71	0.71	0.78	0.78	0.62	0.62
Non-phytate P	0.48	0.48	0.33	0.33	0.44	0.44	0.29	0.29	0.39	0.39	0.24	0.24
Na	0.18	0.18	0.18	0.18	0.18	0.18	0.18	0.18	0.18	0.18	0.18	0.18
Analyzed nutrient composition, % (unless otherwise noted)												
Phytase activity, FTU/kg^5^	< 50	< 50	3,550	3,460	< 50	< 50	4,570	5,020	< 50	< 50	4,620	4,400
Ca	1.03	0.91	0.79	0.77	0.89	0.90	0.63	0.76	0.80	0.84	0.60	0.62
Total P	0.78	0.78	0.59	0.57	0.68	0.61	0.45	0.55	0.60	0.59	0.45	0.40

1 Trace mineral premix include per kg of diet: Mn (manganese sulfate), 120 mg; Zn (zinc sulfate), 100 mg; Fe (iron sulfate monohydrate), 30 mg; Cu (tri-basic copper chloride), 8 mg; I (ethylenediamine dihydriodide), 1.4 mg; and Se (sodium selenite), 0.3 mg.

2 Vitamin premix includes per kg of diet: Vitamin A (Vitamin A acetate), 18,739 IU; Vitamin D_3_ (cholecalciferol), 6,614 IU; Vitamin E (DL-alpha tocopherol acetate), 66 IU; menadione (menadione sodium bisulfate complex), 4 mg; Vitamin B_12_ (cyanocobalamin), 0.03 mg; folacin (folic acid), 2.6 mg: D-pantothenic acid (calcium pantothenate), 31 mg; riboflavin (riboflavin), 22 mg; niacin (niacinamide), 88 mg; thiamin (thiamin mononitrate), 5.5 mg; biotin (biotin), 0.18 mg; and pyridoxine (pyridoxine hydrochloride), 7.7 mg.

3 Econase XT, AB Vista Feed Ingredients, Marlborough, UK.

4 *Escherichia coli-*derived phytase from *Trichoderma reesei* (Quantum Blue 5G, AB Vista Feed Ingredients, Marlborough, UK).

5 One unit of phytase activity (FTU) is the quantity of enzyme to liberate 1 µmol of inorganic phosphate per minute from 0.0051 mol/L sodium phytate at pH 5.5 and 37°C.

### Sample Collections and Chemical Analyses

In Experiment 2, four birds per pen were randomly selected for necropsy at both 28 and 43 d of age. Samples of hypothalamus were collected to determine mRNA expression of orexigenic [neuropeptide Y (**NPY**) and agouti-related peptide (**AGRP**)] and anorexigenic [proopiomelanocortin (**POMC**), cholecystokinin A receptor (**CCKAR**), and ghrelin] peptide hormones. In addition, hypothalamic concentrations of dopamine and serotonin were determined. Hypothalamus samples were collected by first decapitating the bird and then the skull was removed to expose the brain. Next, cuts were made to detach the olfactory nerves and the whole brain was removed. Finally, the hypothalamus was collected into a 1.5-mL tube by referring to a standardized stereotaxic atlas (plate 4.6–5.6), which designated the region of interest as the nucleus infundibuli hypothalami (Kuenzel and Masson, 1988). Samples were immediately frozen by submersion in liquid nitrogen for approximately 5 min to prevent degradation and stored at –80°C until further analysis.

Hypothalamus samples from 2 birds per pen were utilized to determine concentrations of dopamine and serotonin. Tissue samples were prepared in a 1.8-mL microfuge tube by adding 300 µL of perchloric acid (0.1 M) and 2,3-dihydroxybenzoic acid to each tube for every 10 mg sample. Samples were sonicated until completely homogenized, kept on ice for 10 min, and vortexed to mix the homogenate. Samples were centrifuged at 16,000 × *g* for 15 min at 4°C and the supernatant was transferred to a new microfuge tube. The supernatant was centrifuged again with a similar condition to precipitate remaining homogenate. Dopamine and serotonin concentrations were determined using HPLC with amperometric detection according to the method by Yang and Beal (2011). A 2.1 × 250 mm Dionex Acclaim 120 C18 HPLC column (Thermo Fisher Scientific, Waltham, MA) was used with a 3 × 20 mm Supelco Supelguard LC18 guard column (Sigma-Aldrich, St. Louis, MO). The column was eluted at a flow rate of 0.2 mL/min with a mixture of citrate buffer, methanol, and water. An aliquot of the sample was injected into the column with a 25 µL loop. Dopamine and serotonin peaks were integrated with Dionex Chromeleon Chromatography Data System software version 7 (Thermo Fisher Scientific). Dopamine and serotonin concentrations were determined by comparing the results with known standards using a linear least-squares regression.

Messenger RNA expression of hypothalamic NPY, AGRP, POMC, CCKAR, and ghrelin were determined from 1 bird per pen (4 replications per treatment). Samples of hypothalamus were weighed and placed into a 5-mL tube containing 20 µL β-mercaptoethanol and RNA-solv reagent (Omega Bio-tek, Inc., Norcross, GA) to lyse cells and protect mRNA from degradation. Samples were homogenized in 2 rounds of homogenization for 30 s using Tissuemiser homogenizer (Fisher Scientific, Hampton, NH). Subsequently, RNA was separated by transferring the solution through an RNA Homogenizer column (Omega Bio-tek, Inc.) into a collection tube. The column and tube were centrifuged at 10,000 × *g* for 1 min. The solution in the collection tube was kept at room temperature for 5 min prior to RNA extraction. Extraction of total RNA was performed using EZNA Total RNA Kit II (Omega Bio-tek, Inc.) according to the manufacturer's recommendation. Total RNA was quantified using DeNovix DS-II Spectrophotometer (DeNovix Inc., Wilmington, DE) with all samples exhibiting optical density ratios (both 260/280 and 260/320) that were between 1.9 to 2.1, thereby indicating high mRNA purity. Spectral scans ranging from 200 to 400 nm further verified sample purity as all RNA samples produced smooth curves exhibiting 1 peak at 260 nm.

Total RNA was reverse transcribed by mixing mRNA solution with Nucleus Free water, 4 µL of Reaction mix (qScript cDNA Synthesis Kit, Quantabio, Beverly, MA), and 1 µL of reverse transcriptase. Reverse transcription of RNA into cDNA was conducted using Bio-Rad T100 Thermal Cycler (Bio-Rad, Hercules, CA) with a reaction volume of 40 µL. The reaction was performed in a single cycle with the following steps: incubation at 25°C for 5 min followed by 42°C for 30 min and enzyme inactivation at 85°C for 5 min. The cDNA was stored at −20°C until gene expression assay. Master mix for each gene was prepared to a reaction volume of 20 µL by mixing the cDNA with SYBR Green (Bio-Rad), forward and reverse primers (Eurofins Genomics LLC, Louisville, KY; Table 3), and nuclease-free water. Primers were prepared to 10 ng concentrations. Real-Time quantitative PCR was performed using Bio-Rad CFX96 Real-Time PCR Detection System (Bio-Rad) under the following conditions: 1 preincubation step of 3 min at 95°C followed by 40 cycles with each cycle consisting of a melting step of 10 s at 95°C, an annealing step of 10 s at 56°C, and an elongation step of 5 s at 65°C. Each sample was run in triplicate with resulting threshold cycle (**CT**) values obtained by averaging the 3 values. Data were expressed as fold change of the target sample relative to the control sample, normalized to a reference gene (β-actin) calculated using 2^−ΔΔCT^ method (Livak and Schmittgen, 2001).

**Table 3:** Forward and reverse primer sequences used for real-time PCR, Experiment 1.

Gene^1^	Direction	Sequences	Accession No.	Reference
β-actin	Forward	GTCCACCGCAAATGCTTCTAA	NM205518.1	Delp et al., 2017
	Reverse	TGCGCATTTATGGGTTTTGTT		
AGRP	Forward	GGTTCTTCAACGCCTTCTGCTA	AB029443.1	Delp et al., 2017
	Reverse	TTCTTGCCACATGGGAAGGT		
CCKAR	Forward	CATTTGAAAACAGCAGAAGCA	NM001081501.1	El-Kassas et al., 2016
	Reverse	CTGCTGAATGACATCACTTGG		
Ghrelin	Forward	GAAGCACTGCCTAACGAAGACA	NM001001131.1	Yi et al., 2015
	Reverse	GGATGCTGAGAAGGAGAATTCCT		
NPY	Forward	CATGCAGGGCACCATGAG	M87294.1	Delp et al., 2017
	Reverse	CAGCGACAAGGCGAAAGTC		
POMC	Forward	GCCAGACCCCGCTGATG	NM001031098.1	Yi et al., 2015
	Reverse	CTTGTAGGCGCTTTTGACGAT		

1 Abbreviations: AGRP, agouti-related peptide; CCKAR, cholecystokinin A receptor; NPY, neuropeptide Y; POMC, proopiomelanocortin.

At 43 d of age, blood samples (3 mL) were collected from 4 birds per pen via heart puncture to determine plasma inositol concentration. Blood samples were collected into a 4.5-mL heparinized tube (S-Monovette 4.5 mL LH, Sarstedt, Numbrecht, Germany) and placed on ice until centrifugation. Blood samples were centrifuged at 1,643 × *g* for 10 min to separate plasma from the whole blood and stored at –20°C until further analysis. Plasma samples were prepared by mixing with 1 M perchloric acid in a 1:2 ratio (plasma:HClO_4_) to precipitate all protein. Samples were centrifuged at 14,000 × *g* for 10 min to collect the supernatant and were sent to the University of East Anglia School of Biological Sciences in Norwich, England for analysis of inositol concentration using HPLC with pulsed amperometric detection. Samples were diluted 50-fold in 18.2 mohm × cm water. An aliquot (20 µL) was injected into a 4 mm × 250 mm MetroSep Carb 2 (Metrohm, Runcorn, UK) HPLC column. The column was eluted at a flow rate of 0.5 mL/min with 150 mM NaOH. Another aliquot (5 µL) was injected onto a 2 mm × 100 mm Metrosep Carb 2 (Metrohm) column with a guard column eluted at a flow rate of 0.2 mL/min with the same solvent. Inositol peaks were integrated with Chromeleon (ThermoFisher Scientific) and DataApex Clarity (DataApex, Prague, Czech Republic) software packages. Inositol concentration was determined by comparing results with known standards using a linear least-squared regression.

#### Statistical Analyses

Experiment 2 was conducted as a randomized complete block design with pen location as the blocking factor. Individual pen represented the experimental unit with 8 replications. Data were analyzed as 2 × 2 factorial treatment arrangement with 2 factors of nutrient content (without or with increased nutrient density) and 2 factors of phytase supplementation (0 or 4,500 FTU/kg). Interactive and main effects were analyzed using 2-way ANOVA by MIXED procedure of SAS (2011) with the following mixed-effect model:

$$Y_{ij}=\mu ..+\tau_{i}+\beta_{j}+{\left(\tau \beta \right)}_{ij}+\varepsilon_{ijk}$$

where μ..is the overall mean; the $\tau_{i}\;$are fixed factor effects of i^th^ level of nutrient content factor (without or with increased nutrient density); the $\beta_{j}$ are fixed factor effects of j^th^ level of phytase inclusion factor (0 or 4500 FTU/kg); the ${\left(\tau \beta \right)}_{ij}$ are interaction effects between the i^th^ level of nutrient content factor and the j^th^ level of phytase inclusion factor; and the $\varepsilon_{ijk}$ are identically and independently normally distributed random errors with mean 0 and a variance $\sigma^{2}$. Means were separated by Tukey's HSD test when a significant interaction effect was observed. Preplanned orthogonal contrasts were conducted between Treatment 1 (control diet) vs. 3 (diet with phytase addition), Treatment 2 (diet with increased nutrient density) vs. 4 (diet with increased nutrient density and phytase addition), and Treatment 3 (diet with phytase addition) vs. 4 (diet with increased nutrient density and phytase addition). However, fold change calculation on mRNA expression of peptide hormones using 2^−ΔΔCT^ method prevented the use of contrast analysis. Hence, only interactive and main effects on mRNA expression data were presented. Statistical significance was considered at *P* ≤ 0.05.

## RESULTS

In Experiment 1, analyzed phytase activity in Treatment 2 was 82.0% of the calculated values based on 4,810 FTU/g analyzed phytase source (Table 1). In Experiment 2, analyzed phytase activity in Treatments 3 and 4 of starter diets were 78.9 and 76.9% of the calculated values (Table 2). The lower analyzed phytase activity may be associated with unexpected lower phytase activity in the phytase source used for Treatments 3 and 4 of in starter diets. Phytase source used in Treatments 3 and 4 of Experiment 2 was the same phytase used in Treatment 2 of Experiment 1 (analyzed as 4,810 FTU/g). However, phytase activity in the phytase source decreased to 3,810 FTU/kg, which may be the reason for the lower phytase activity in Treatments 3 and 4 of starter diets in Experiment 2. Therefore, the inclusion of phytase for Treatments 3 and 4 of grower and finisher diets in Experiment 2 was increased to compensate for the lower phytase activity in the phytase source. This resulted in similar analyzed phytase activity in Treatments 3 and 4 of grower and finisher diets compared with the calculated values.

### Experiment 1

Broilers fed the diet formulated with phytase addition at 4,500 FTU/kg had higher (*P* < 0.05) BW gain (0.782 vs. 0.749 kg/bird) and feed intake (0.936 vs. 0.909 kg/bird) compared with birds fed the control diet from 1 to 19 d of age. However, FCR of broilers receiving the diet with phytase addition (1.199) was similar (*P* = 0.22) with those fed the control diet (1.215). In addition, broilers fed the control diet (2,809 kcal/kg) had lower (*P* < 0.001) AME_n_ than birds fed the phytase-supplemented diet (2,872 kcal/kg). Apparent ileal digestibility of Arg, Ile, Leu, Asp, Glu, and Pro in the diet formulated with phytase inclusion was higher (*P* < 0.05) than in the control diet (Table 4).

**Table 4:** Apparent pre-cecal digestibility (%) of indispensable and dispensable amino acids in diets fed to broilers without or with phytase supplementation from 1 to 23 D of age, Experiment 1^1^.

	Dietary treatments			
Amino acids	1	2^2^	SEM^3^	*P*-value
Indispensable				
Arg	89.69	91.23	0.46	0.033
Cys	73.37	74.40	1.55	0.65
His	86.01	87.14	0.65	0.24
Ile	82.14	84.95	0.88	0.042
Leu	83.11	85.79	0.78	0.029
Lys	88.56	89.77	0.49	0.10
Met	93.73	94.45	0.34	0.16
Phe	84.59	86.72	0.72	0.06
Thr	79.64	81.05	0.82	0.24
Trp	79.65	81.05	0.91	0.29
Val	82.52	84.67	0.82	0.08
Dispensable				
Ala	83.60	85.72	0.75	0.07
Asp	82.23	84.26	0.66	0.047
Glu	87.63	89.79	0.52	0.011
Gly	79.87	81.20	0.83	0.19
Pro	84.05	86.50	0.70	0.027
Ser	82.08	84.11	0.77	0.08
Tyr	82.91	84.55	0.77	0.16

1 Values are least-square means of 16 replicate cages with 8 birds per cage at placement.

2 Treatment 2 was formulated with the addition of phytase at 4,500 FTU/kg. One unit of phytase activity (FTU) is the quantity of enzyme to liberate 1 µmol of inorganic phosphate per minute from 0.0051 mol/L sodium phytate at pH 5.5 and 37°C.

3 Pooled standard error of the mean.

### Experiment 2

No interactive effects (*P* > 0.05) between nutrient density and phytase supplementation were observed on feed intake and FCR of broilers from 1 to 14 d of age (Table 5). However, preplanned orthogonal contrasts indicated that broilers consuming diets formulated with the combination of increased nutrient density and phytase inclusion (Treatment 4) had 5 and 6% higher (*P* < 0.05) BW gain than birds fed diets with either increased nutrient density (Treatment 2) or phytase addition (Treatment 3), respectively. Phytase addition in Treatment 3 decreased (*P* = 0.013) FCR of broilers by 6 points compared with broilers provided the control diet (Treatment 1). Nutrient density and phytase inclusion main effects had positive influence (*P* < 0.05) on BW gain and FCR of broilers. Incidence of mortality was higher (*P* = 0.034) in broilers provided diets with increased nutrient density than those without nutrient density. Interestingly, 75% of mortality of birds provided diets with increased nutrient density occurred within 7 d post-hatch. The reason for the higher incidence of mortality is unknown.

**Table 5:** Growth performance of broilers fed diets enhanced with increased nutrient density and/or phytase supplementation from 1 to 14 D of age, Experiment 2^1^.

Treatment	Nutrient density^2^	Phytase, FTU/kg^3^	BW,kg/bird	BW gain, kg/bird	Feed intake, kg/bird	FCR,kg:kg^4^	Mortality,%^5^
1	−	0	0.398	0.354	0.452	1.276	0.0
2	+	0	0.410	0.367	0.442	1.207	1.9
3	−	4,500	0.406	0.363	0.440	1.215	0.6
4	+	4,500	0.428	0.385	0.453	1.178	1.5
SEM^6^			0.006	0.006	0.007	0.017	0.8
Main effect of nutrient density							
	−		0.402	0.359	0.446	1.245	0.3
	+		0.419	0.376	0.448	1.193	1.7
	SEM^6^		0.005	0.005	0.005	0.012	0.6
Main effect of phytase							
		0	0.404	0.361	0.447	1.241	1.0
		4,500	0.417	0.374	0.446	1.197	1.0
		SEM^6^	0.004	0.004	0.005	0.012	0.6
Source of variation				*Probabilities*			
	
Nutrient density × phytase			0.349	0.325	0.099	0.303	0.802
Treatment 1 vs. 3			0.242	0.257	0.224	0.013	0.540
Treatment 2 vs. 4			0.012	0.011	0.244	0.159	0.754
Treatment 3 vs. 4			0.006	0.006	0.195	0.101	0.176
Main effect of nutrient density			0.002	0.002	0.832	0.002	0.034
Main effect of phytase			0.013	0.013	0.892	0.007	0.507

1 Values are least-square means of 8 replicate pens with each pen having 26 birds at placement.

2 − = without increased nutrient density, + = with increased nutrient density; diets with increased nutrient density were formulated to contain 0.007, 0.015, 0.013, 0.021, 0.024%, and 61 kcal/kg higher digestible SAA, Lys, Thr, Val, Ile concentrations, and AME_n_ (obtained from Experiment 1), respectively, compared with the control diet.

3 One unit of phytase activity (FTU) is the quantity of enzyme to liberate 1 µmol of inorganic phosphate per minute from 0.0051 mol/L sodium phytate at pH 5.5 and 37°C.

4 Feed conversion ratio (feed intake/BW gain) data were corrected for mortality.

5 Mortality data were arcsine transformed.

6 Pooled standard error of the mean.

From 1 to 28 d of age, the combination of increased nutrient density and phytase addition (Treatment 4) resulted in broilers having higher (*P* < 0.05) feed intake than birds fed diets with increased nutrient density (Treatment 2; 2.148 vs. 2.030 kg) or phytase inclusion only (Treatment 3; 2.148 vs. 2.033 kg) (Table 6). Pre-planned orthogonal contrasts detected that broilers provided diets with the combination of increased nutrient density and phytase supplementation (Treatment 4) had higher (*P* < 0.01) BW gain than those fed diets with either increased nutrient density (Treatment 2) or phytase addition (Treatment 3). Feed conversion of broilers supplemented with phytase (Treatment 3) was 3.7 points lower (*P* = 0.049) compared with broilers fed the control diet (Treatment 1). Main effects of nutrient density demonstrated benefits in increasing (*P* < 0.001) BW gain and decreasing (*P* = 0.004) FCR of broilers by 5.3 and 2.9% compared with those receiving the control diet. Similarly, BW gain of broilers was 3.8% higher (*P* = 0.010) in broilers fed diets supplemented with phytase compared with those fed the control diet. The incidence of mortality was not impacted (*P* > 0.05) by dietary treatments.

**Table 6:** Growth performance of broilers fed diets enhanced with increased nutrient density and/or phytase supplementation from 1 to 28 D of age, Experiment 2^1^.

Treatment	Nutrient density^2^	Phytase, FTU/kg^3^	BW,kg/bird	BW gain, kg/bird	Feed intake, kg/bird	FCR,kg:kg^4^	Mortality,%^5^
1	−	0	1.552	1.509	2.052^ab^	1.360	3.2
2	+	0	1.604	1.561	2.030^b^	1.301	6.5
3	−	4,500	1.581	1.538	2.033^ab^	1.323	4.7
4	+	4,500	1.690	1.647	2.148^a^	1.304	2.6
SEM^6^			0.021	0.021	0.031	0.013	1.4
Main effect of nutrient density							
	−		1.567	1.523	2.042	1.341	4.0
	+		1.647	1.604	2.089	1.302	4.6
	SEM^6^		0.015	0.015	0.022	0.009	1.1
Main effect of phytase							
		0	1.578	1.535	2.041	1.330	3.7
		4,500	1.636	1.593	2.090	1.314	3.2
		SEM^6^	0.015	0.015	0.022	0.009	1.4
Source of variation				*Probabilities*			
	
Nutrient density × phytase			0.188	0.185	0.033	0.113	0.099
Treatment 1 vs. 3			0.327	0.329	0.664	0.049	0.494
Treatment 2 vs. 4			0.006	0.006	0.010	0.829	0.093
Treatment 3 vs. 4			0.001	0.001	0.015	0.308	0.396
Main effect of nutrient density			< 0.001	< 0.001	0.135	0.004	0.639
Main effect of phytase			0.010	0.010	0.112	0.194	0.480

a-b Means not sharing a common superscript within column differ (*P* < 0.05).

1 Values are least-square means of 8 replicate pens with each pen having 26 birds at placement.

2 − = without increased nutrient density, + = with increased nutrient density; diets with increased nutrient density were formulated to contain 0.007, 0.015, 0.013, 0.021, 0.024%, and 61 kcal/kg higher digestible SAA, Lys, Thr, Val, Ile concentrations, and AME_n_ (obtained from Experiment 1), respectively, compared with the control diet.

3 One unit of phytase activity (FTU) is the quantity of enzyme to liberate 1 µmol of inorganic phosphate per minute from 0.0051 mol/L sodium phytate at pH 5.5 and 37°C.

4 Feed conversion ratio (feed intake/BW gain) data were corrected for mortality.

5 Mortality data were arcsine transformed.

6 Pooled standard error of the mean.

From 1 to 40 d of age, interactive effects were reflected in the FCR of broilers where broilers consuming diets supplemented with the combination of increased nutrient density and phytase supplementation had 3.3% lower (*P* = 0.025) FCR compared with birds fed diets with phytase inclusion only (Table 7). Furthermore, preplanned orthogonal contrasts indicated increased (*P* < 0.05) BW gain by 3.6 and 4.4% when broilers were fed diets with both increased nutrient density and phytase inclusion (Treatment 4) compared with birds fed diets either with increased nutrient density (Treatment 2) or phytase addition (Treatment 3), respectively. Main effects of nutrient density demonstrated an increase (*P* = 0.011) in BW gain of broilers by 3.3% compared with broilers provided diets without nutrient density. In contrast, main effects of phytase addition on increasing feed intake (*P* = 0.06) and BW gain (*P* = 0.051) approached significance compared with birds fed diets without phytase inclusion. No influence (*P* > 0.05) of dietary treatments was observed on the incidence of mortality.

**Table 7:** Growth performance of broilers fed diets enhanced with increased nutrient density and/or phytase supplementation from 1 to 40 D of age, Experiment 2^1^.

Treatment	Nutrient density^2^	Phytase, FTU/kg^3^	BW,kg/bird	BW gain, kg/bird	Feed intake, kg/bird	FCR,kg:kg^4^	Mortality,%^5^
1	−	0	2.930	2.887	4.176	1.447^ab^	5.4
2	+	0	2.993	2.950	4.294	1.456^ab^	6.5
3	−	4,500	2.967	2.925	4.310	1.475^a^	4.7
4	+	4,500	3.104	3.055	4.356	1.426^b^	3.6
SEM^6^			0.035	0.036	0.054	0.014	1.4
Main effect of nutrient density							
	−		2.948	2.906	4.243	1.461	5.0
	+		3.049	3.003	4.325	1.441	5.0
	SEM^6^		0.025	0.025	0.039	0.010	1.0
Main effect of phytase							
		0	2.962	2.918	4.235	1.451	6.0
		4,500	3.035	2.990	4.333	1.450	4.1
		SEM^6^	0.024	0.025	0.038	0.010	1.0
Source of variation				*Probabilities*			
	
Nutrient density × phytase			0.283	0.349	0.483	0.025	0.513
Treatment 1 vs. 3			0.465	0.455	0.084	0.125	0.246
Treatment 2 vs. 4			0.024	0.037	0.370	0.081	0.756
Treatment 3 vs. 4			0.008	0.015	0.521	0.009	0.546
Main effect of nutrient density			0.006	0.011	0.117	0.109	0.841
Main effect of phytase			0.039	0.051	0.064	0.948	0.288

a-b Means not sharing a common superscript within column differ (*P* < 0.05).

1 Values are least-square means of 8 replicate pens with each pen having 26 birds at placement.

2 − = without increased nutrient density, + = with increased nutrient density; diets with increased nutrient density were formulated to contain 0.007, 0.015, 0.013, 0.021, 0.024%, and 61 kcal/kg higher digestible SAA, Lys, Thr, Val, Ile concentrations, and AME_n_ (obtained from Experiment 1), respectively, compared with the control diet.

3 One unit of phytase activity (FTU) is the quantity of enzyme to liberate 1 µmol of inorganic phosphate per minute from 0.0051 mol/L sodium phytate at pH 5.5 and 37°C.

4 Feed conversion ratio (feed intake/BW gain) data were corrected for mortality.

5 Mortality data were arcsine transformed.

6 Pooled standard error of the mean.

Carcass, total breast meat, and thigh weights of broilers provided diets with the combination of increased nutrient density and phytase were heavier (*P* < 0.05) among dietary treatments (Table 8). Preplanned orthogonal contrasts revealed that the combination of increased nutrient density and phytase addition led to greater (*P* < 0.05) weights of carcass, total breast meat, wings, drums, and thighs of broilers compared with birds fed diets with either increased nutrient density or phytase supplementation alone. Similar response was also observed on carcass and total breast meat of broilers provided the combination of increased nutrient density and phytase inclusion having greater yields (*P* < 0.05) relative to live weight compared with consuming diets formulated with increased nutrient density. Thigh yield of broilers fed the combination of increased nutrient density and phytase addition (Treatment 4) diets was also higher (*P* < 0.05) than those of broilers fed diets either with increased nutrient density (Treatment 2) or phytase inclusion (Treatment 3). Main effects of nutrient density were apparent in increasing (*P* < 0.05) wing weight, thigh yield, and abdominal fat weight. Likewise, main effects of phytase supplementation increased (*P* < 0.001) carcass yield and drum weight of broilers.

**Table 8:** Carcass characteristics of broilers fed diets with increased nutrient density and/or phytase supplementation from 1 to 41 D of age, Experiment 2^1^.

			Carcass		Total breast		Wing		Drum		Thigh		Abdominal fat	
Treatment	Nutrient density^2^	Phytase, FTU/kg^3^	Weight, g	Yield, %^4^	Weight, g	Yield, %^4^	Weight, g	Yield, %^4^	Weight, g	Yield, %^4^	Weight, g	Yield,%^4^	Weight, g	Percent, %^4^
1	−	0	2,249^b^	74.60	795^b^	26.38	224	7.43	265	8.79	281^b^	9.33	36	1.21
2	+	0	2,259^b^	74.23	784^b^	25.75	229	7.51	270	8.89	287^b^	9.42	39	1.27
3	−	4,500	2,269^b^	74.78	805^b^	26.41	228	7.53	269	8.88	285^b^	9.40	35	1.17
4	+	4,500	2,376^a^	74.84	838^a^	26.38	239	7.54	282	8.90	305^a^	9.62	38	1.21
	SEM^5^		19	0.28	9	0.20	2	0.04	3	0.05	4	0.09	1	0.04
Main effect of nutrient density														
	−		2,259	74.69	800	26.39	226	7.48	227	8.84	283	9.36	36	1.19
	+		2,317	74.54	811	26.07	234	7.52	276	8.89	296	9.52	39	1.24
	SEM^5^		14	0.24	6	0.14	1	0.03	2	0.04	4	0.07	1	0.03
Main effect of phytase														
		0	2,254	74.42	790	26.06	226	7.47	267	8.84	284	9.38	37	1.24
		4,500	2,322	74.81	821	26.40	234	7.53	276	8.89	295	9.51	37	1.19
		SEM^5^	13	0.24	6	0.14	1	0.03	2	0.04	4	0.07	1	0.03
Source of variation							*Probabilities*							
	
Nutrient density × phytase			0.005	0.237	0.009	0.117	0.091	0.271	0.094	0.404	0.018	0.362	0.718	0.713
Treatment 1 vs. 3			0.427	0.474	0.471	0.895	0.066	0.057	0.165	0.175	0.375	0.523	0.544	0.382
Treatment 2 vs. 4			< 0.001	0.012	< 0.001	0.014	< 0.001	0.653	< 0.001	0.808	< 0.001	0.041	0.901	0.135
Treatment 3 vs. 4			< 0.001	0.822	0.006	0.916	< 0.001	0.931	< 0.001	0.788	< 0.001	0.028	0.029	0.320
Main effect of nutrient density			< 0.001	0.385	0.206	0.085	< 0.001	0.220	< 0.001	0.225	< 0.001	0.028	0.006	0.076
Main effect of phytase			< 0.001	0.026	< 0.001	0.078	< 0.001	0.088	< 0.001	0.245	< 0.001	0.064	0.596	0.098

a-b Means not sharing a common superscript within column differ (*P* < 0.05).

1 Values are least-square means of 8 replicate pens with 14 birds selected from each pen for processing.

2 − = without increased nutrient density, + = with increased nutrient density; diets with increased nutrient density were formulated to contain 0.007, 0.015, 0.013, 0.021, 0.024%, and 61 kcal/kg higher digestible SAA, Lys, Thr, Val, Ile concentrations, and AME_n_ (obtained from Experiment 1), respectively, compared with the control diet.

3 One unit of phytase activity (FTU) is the quantity of enzyme to liberate 1 µmol of inorganic phosphate per minute from 0.0051 mol/L sodium phytate at pH 5.5 and 37°C.

4 Percent yield was calculated based on weight proportion to bird live weight at 40 d of age.

5 Pooled standard error of the mean.

Hypothalamic mRNA expression of AGRP, CCKAR, ghrelin, NPY, and POMC at 28 and 43 d of age were not influenced (*P* > 0.05) by dietary treatments (Table 9). Additionally, there were no interactive effects of dietary treatments and main effects of nutrient density (*P* > 0.05) on hypothalamic concentrations of dopamine or serotonin at either 28 or 43 d of age (Table 10). However, phytase supplementation increased (*P* = 0.008) hypothalamic dopamine by 20% at 43 d of age. Phytase inclusion also increased (*P <* 0.001) plasma inositol of broilers by 2.3-fold compared with birds fed diets without phytase supplementation.

**Table 9:** Hypothalamic appetitive hormone expression in broilers fed diets enhanced with increased nutrient density and/or phytase supplementation, Experiment 2^1^.

			28 D of age					43 D of age				
Treatment	Nutrient density^2^	Phytase, FTU/kg^3^	AGRP^4^	CCKAR^4^	Ghrelin	NPY^4^	POMC^4^	AGRP^4^	CCKAR^4^	Ghrelin	NPY^4^	POMC^4^
1	−	0	1.094	1.154	1.304	1.114	1.097	0.889	1.082	1.118	1.173	1.089
2	+	0	1.070	1.466	0.902	1.257	0.523	1.160	1.103	2.543	1.082	0.666
3	−	4,500	1.028	1.261	0.864	0.873	1.208	1.402	1.629	2.194	1.372	1.537
4	+	4,500	0.977	1.612	0.947	1.106	1.352	1.129	1.365	2.311	1.275	1.242
SEM^5^			0.347	0.470	0.370	0.312	0.277	0.574	0.311	0.673	0.318	0.256
Main effect of nutrient density												
	−		1.332	1.079	1.336	0.726	1.091	1.138	1.103	1.271	1.174	1.106
	+		1.285	1.401	1.140	0.855	0.888	1.104	1.004	1.863	1.088	0.803
	SEM^5^		0.355	0.312	0.410	0.155	0.222	0.338	0.213	0.357	0.220	0.164
Main effect of phytase												
		0	1.099	1.068	1.335	1.568	1.267	1.137	1.134	1.250	1.118	1.155
		4,500	1.018	1.223	1.096	0.723	2.003	1.400	1.553	1.538	1.313	1.829
		SEM^5^	0.287	0.288	0.402	0.544	0.352	0.379	0.264	0.328	0.235	0.239
Source of variation						*Probabilities*						
					
Nutrient density × phytase			0.990	0.778	0.527	0.765	0.134	0.925	0.394	0.477	0.901	0.166
Main effect of nutrient density			0.882	0.295	0.497	0.465	0.454	0.944	0.644	0.260	0.733	0.211
Main effect of phytase			0.752	0.597	0.395	0.263	0.083	0.633	0.103	0.545	0.470	0.067

1 Values represent the fold change of appetitive hormone expressions in a target sample relative to the control sample, normalized to a reference gene (β-actin) from 4 replicate pens with 1 bird per pen. These values were calculated using 2^−ΔΔCT^ method (Livak and Schmittgen, 2001).

2 −= without increased nutrient density, + = with increased nutrient density; diets with increased nutrient density were formulated to contain 0.007, 0.015, 0.013, 0.021, 0.024%, and 61 kcal/kg higher digestible SAA, Lys, Thr, Val, Ile concentrations, and AME_n_ (obtained from Experiment 1), respectively, compared with the control diet.

3 One unit of phytase activity (FTU) is the quantity of enzyme to liberate 1 µmol of inorganic phosphate per minute from 0.0051 mol/L sodium phytate at pH 5.5 and 37°C.

4 Abbreviations: AGRP, agouti-related neuropeptide, CCKAR, cholecystokinin A receptor, NPY, neuropeptide Y, POMC, proopiomelanocortin.

5 Pooled standard error of the mean.

**Table 10:** Hypothalamic catecholamine (ng/mg) and plasma inositol concentrations (µM) of broilers fed diets with increased nutrient density and/or phytase supplementation, Experiment 2^1^.

			28 D of age		43 D of age		
Treatment	Nutrient density^2^	Phytase, FTU/kg^3^	Dopamine	Serotonin	Dopamine	Serotonin	Plasma inositol
1	−	0	0.779	0.784	0.494	0.340	179
2	+	0	0.708	0.815	0.549	0.404	169
3	−	4,500	0.670	0.758	0.608	0.409	403
4	+	4,500	0.715	0.793	0.645	0.453	407
SEM^4^			0.064	0.097	0.040	0.063	23
Main effect of nutrient density							
	−		0.724	0.771	0.551	0.374	291
	+		0.711	0.804	0.597	0.428	288
	SEM^4^		0.048	0.069	0.031	0.045	16
Main effect of phytase							
		0	0.743	0.799	0.521	0.372	174
		4,500	0.693	0.775	0.627	0.431	405
		SEM^4^	0.048	0.069	0.031	0.045	16
Source of variation				*Probabilities*			
	
Nutrient density × phytase			0.334	0.985	0.796	0.875	0.748
Treatment 1 vs. 3			0.205	0.850	0.034	0.448	< 0.001
Treatment 2 vs. 4			0.929	0.871	0.071	0.590	< 0.001
Treatment 3 vs. 4			0.594	0.801	0.481	0.628	0.895
Main effect of nutrient density			0.826	0.736	0.216	0.402	0.892
Main effect of phytase			0.399	0.804	0.008	0.361	< 0.001

1 Values are least-square means of 8 replicate pens with 2 birds per pen for catecholamine analysis and average of 4 birds per pen for plasma inositol analysis.

2 − = without increased nutrient density, + = with increased nutrient density; diets with increased nutrient density were formulated to contain 0.007, 0.015, 0.013, 0.021, 0.024%, and 61 kcal/kg higher digestible SAA, Lys, Thr, Val, Ile concentrations, and AME_n_ (obtained from Experiment 1), respectively, compared with the control diet.

3 One unit of phytase activity (FTU) is the quantity of enzyme to liberate 1 µmol of inorganic phosphate per minute from 0.0051 mol/L sodium phytate at pH 5.5 and 37°C.

4 Pooled standard error of the mean.

## DISCUSSION

This study examined the response of broilers to phytase supplementation and increased nutrient density on growth performance, meat yield, and hypothalamic appetitive hormone expression and catecholamine concentrations. In Experiment 1, benefits of phytase inclusion in broiler diets were evident in the enhancement of nutrient availability (Selle and Ravindran, 2007). Gehring et al. (2013) reported that phytase supplementation at 2,000 FTU/kg was beneficial in increasing the digestibility of amino acids in broilers compared with birds fed diets without phytase addition from 27 to 32 d of age. The observed increase in amino acid digestibility in broilers fed phytase-added diets may be attributed to the influence of phytase in hindering protein-phytate interactions (Selle et al., 2012). This mechanism concomitantly decreases mucin secretion and endogenous amino acid losses (Cowieson et al., 2004). Similarly, the degradation of phytate by phytase may also increase AME_n_ through direct liberation of starch (Rickard and Thompson, 1997). Phytase supplementation from 0 to 12,000 FTU/kg was reported to quadratically increased AME_n_ of broilers (Shirley and Edwards, 2003).

Experiment 2 indicated interactive effects of phytase and nutrient density on decreased FCR and improved carcass characteristics of broilers. In agreement, Selle et al. (2007) observed an increase in BW gain and a reduction in FCR of broilers when birds were fed diets with the combination of phytase supplementation and increasing digestible Lys concentration from 1 to 21 d of age. Data from the current experiment indicated that birds were responding to the additional nutrients regardless of whether they were released by the phytase or added directly to the ration. Recent investigation indicated that phytase supplementation of *E. coli-*derived phytase from *Trichoderma reesei* at 4,000 FTU/kg increased mRNA expression of system L amino acid transporter 4 and sodium-coupled neutral amino acid transporter 1 in the jejunum of broilers at 18 d of age (Walk and Olukosi, 2019). The increased amino acid transporters due to phytase supplementation may allow for an increased nutrient uptake when provided diets with increased nutrient density. Furthermore, the greater nutrient concentrations in those diets with increased nutrient density may allow the increased supply of amino acids and energy for enhanced muscle accretion (Wu, 2014; Barzegar et al., 2020), which was also promoted by the effect of phytase supplementation. The similarity in gain and FCR responses to the increased nutrient density or the phytase alone indicated that the estimated phytase effects applied via increased nutrient density determined in Experiment 1 were reasonably accurate.

In the current research, phytase inclusion was observed to increase plasma inositol concentration. Inositol has been reported to increase protein synthesis and muscle accretion through the activation of calmodulin/calcineurin A (Tokomitsu et al., 1999; McKinsey et al., 2002) and Akt/mTOR pathways (Hassan et al., 2013). Schmeisser et al. (2017) indicated that supplementing bacterial phytase from *Citrobacter braakii* at 1,000 FTU/kg upregulated mRNA expression of phosphatidylinositide-3-phosphate kinase and myocyte enhancer factors 2A and A, which are involved with protein synthesis in the *pectoralis major* muscle of broilers. Broilers receiving phytase supplementation at 1,000 FTU/kg had heavier breast meat weight compared with birds fed diets without phytase inclusion. Hence, the liberation of inositol from phytate coupled with greater nutrient release by phytase may have a combined effect in enhancing muscle accretion of phytase-supplemented broilers.

In the present research, interactive effects of increased nutrient density and phytase supplementation were observed on increased total feed intake of broilers from 1 to 28 d of age. The mechanism of this interactive effect on feed intake of broilers is unsure. However, it is possible that the increased feed intake may be simply the result of greater BW gain of broilers due to the combined effects of increased nutrient density and phytase supplementation. It is also interesting that phytase supplementation numerically (*P* = 0.06) increased feed intake of broilers compared with broilers fed diets without phytase inclusion. The numerical increase of feed intake is still worth noting as it may indicate a biological response of phytase on feed intake stimulation. It is difficult to explain the reason for the insignificant feed intake response as previous research in our laboratory indicated increased BW gain and feed intake from 1 to 40 d of age (r = 0.73, *P* < 0.001) when broilers were fed diets with increasing *E. coli-*derived phytase supplementation up to 40,500 FTU/kg (Kriseldi et al., 2019).

In addition to the work by Kriseldi et al. (2019), extra-phosphoric effects of phytase on feed intake stimulation have been previously reported. Walk and Olukosi (2019) also noted that increased BW gain of broilers from 1 to 28 d of age due to phytase supplementation was correlated (r = 0.33 to 0.72, *P* < 0.10) with digestible amino acid intake but not (r = –0.29 to 0.14, *P >* 0.10) with apparent ileal digestibility of amino acids. In addition, previous research reported that additions of dietary phytase ranging from 1,500 to 4,000 FTU/kg enhanced feed intake of broilers by 22 and 207 g from 1 to 14 and 1 to 42 d of age (Walk et al., 2012, 2014; Gehring et al., 2013; Campasino et al., 2014; Beeson et al., 2017; Lee et al., 2019; Walk and Olukosi, 2019).

The mechanism of feed intake stimulation through phytase supplementation is not well elucidated in the literature. Possibly, stimulation of feed intake is associated with the role of phytase on phytate degradation (Liu et al., 2014). A previous study demonstrated that grass carp provided diets with phytic acid supplementation at 0.4% had higher mRNA expression of CCK, cocaine- and amphetamine-regulated transcript (**CART**), and ghrelin in the brain, which led to lower feed intake compared with those fed diets without phytic acid addition (Liu et al., 2014). Cholecystokinin has been reported to promote satiety through CCKAR by inhibiting mRNA expression of NPY and AGRP (Bi et al., 2004; Chen et al., 2008; Dunn et al., 2013) and stimulating mRNA expression of POMC (Fan et al., 2004). Similarly, CART inhibits feed intake in poultry by lowering the expression of NPY (Tachibana et al., 2003). In addition, ghrelin in avian species has been reported as a suppressor of appetite mediated by corticotropin-releasing factor (Kaiya et al., 2007).

Conversely, the hydrolysis of phytate by phytase supplementation may increase feed intake of broilers through increasing digesta passage rate (Watson et al., 2006). Presumably, the higher digesta passage rate may reduce CCK secretion (Scanes and Pierzchala-Koziec, 2014) and decrease the inhibition of NPY and AGRP in the hypothalamus (Bi et al., 2004; Chen et al., 2008; Dunn et al., 2013). However, hypothalamic mRNA expression of orexigenic (AGRP and NPY) and anorexigenic (CCKAR, ghrelin, and POMC) appetite hormones in the current research were not influenced by dietary treatments. The reason for the lack of differences in hypothalamic appetitive hormone expression may be attributed to variation within each treatment. Hence, additional data are warranted to determine the role of phytase on changes in appetite hormone concentrations.

The current research evaluated hypothalamic dopamine concentration as an indicator of phytase effects on changes in feed intake. Data in the study presented herein demonstrated that phytase supplementation resulted in increased dopamine concentration in the hypothalamus of broilers. However, dopamine concentration was not correlated (r = 0.25, *P* = 0.17) with feed intake of broilers from 1 to 40 d of age. Similarly, Bungo et al. (2010) observed no response of intra-cerebroventricular injection of dopamine on feed intake of broilers after 60 min of injection. In contrast, Zendehdel et al. (2019) reported increased feed intake of broilers within 120 min following intra-cerebroventricular injection of dopamine 1 and 2 receptors antagonists.

It is possible that the increased dopamine concentration was affected by the inositol released from phytase supplementation. A recent study reported that broilers fed diets with inositol supplementation at 0.38 and 0.35% from 1 to 11 and 12 to 22 d of age, respectively, had higher plasma dopamine concentration compared with broilers fed diets devoid of supplemental inositol (Gonzalez-Uarquin et al., 2020). However, the concentration of dopamine in the plasma may not necessarily correlate with the concentration in the brain. For example, microorganisms may contribute to the concentration of dopamine in the periphery through the production of short chain fatty acids, which can alter the enzyme required for dopamine production (Lyte and Lyte, 2019). This was evident as the concentration of plasma inositol of broilers in the present study had only a weak correlation with hypothalamic dopamine concentration (r = 0.37, *P* = 0.038). Hence, other mode of actions, such as stress level, may influence the response of hypothalamic dopamine to phytase supplementation in broilers (Herwig et al., 2019).

The elevated dopamine concentration may play a role in muscle accretion. Previous research indicated that the activation of dopamine 1 and 5 receptors with dopamine receptor agonists increased *tibialis anterior* and *medial gastrocnemius* muscle mass in mice (Reichart et al., 2011). This positive effect may be attributed to the increased activity of cyclic adenosine monophosphate in those muscles (Reichart et al., 2011), which allows for the activation of protein kinase A leading to muscle hypertrophy (Berdeaux and Stewart, 2012). This mechanism may have contributed to the effect of phytase on enhancing carcass characteristics in the present research. However, this mechanism has not been elucidated in birds.

This research demonstrated additive effects of phytase supplementation and nutrient density on enhanced growth performance and carcass characteristics of broilers. This attribute may be due to synergistic effects of increased nutrient density in providing amino acids and energy as building blocks of muscle accretion and phytase in increasing protein synthesis via inositol liberation and hypothalamic dopamine concentration. However, this study could not confirm the potential extra-phosphoric effects of phytase through the stimulation of feed intake although numerical increase of feed intake was observed. Therefore, additional data are warranted to determine extra-phosphoric effects of phytase on feed intake stimulation.
